# Determinants of Maternity Care Services Utilization among Married Adolescents in Rural India

**DOI:** 10.1371/journal.pone.0031666

**Published:** 2012-02-15

**Authors:** Prashant Kumar Singh, Rajesh Kumar Rai, Manoj Alagarajan, Lucky Singh

**Affiliations:** 1 International Institute for Population Sciences, Deonar, Mumbai, India; 2 Braun School of Public Health and Community Medicine, Hebrew University-Hadassah, Jerusalem, Israel; 3 Department of Development Studies, International Institute for Population Sciences, Deonar, Mumbai, India; Aga Khan University, Pakistan

## Abstract

**Background:**

Coupled with the largest number of maternal deaths, adolescent pregnancy in India has received paramount importance due to early age at marriage and low contraceptive use. The factors associated with the utilization of maternal healthcare services among married adolescents in rural India are poorly discussed.

**Methodology/Principal Findings:**

Using the data from third wave of National Family Health Survey (2005–06), available in public domain for the use by researchers, this paper examines the factors associated with the utilization of maternal healthcare services among married adolescent women (aged 15–19 years) in rural India. Three components of maternal healthcare service utilization were measured: full antenatal care, safe delivery, and postnatal care within 42 days of delivery for the women who gave births in the last five years preceding the survey. Considering the framework on causes of maternal mortality proposed by Thaddeus and Maine (1994), selected socioeconomic, demographic, and cultural factors influencing outcome events were included as the predictor variables. Bi-variate analyses including chi-square test to determine the difference in proportion, and logistic regression to understand the net effect of predictor variables on selected outcomes were applied. Findings indicate the significant differences in the use of selected maternal healthcare utilization by educational attainment, economic status and region of residence. Muslim women, and women belonged to Scheduled Castes, Scheduled Tribes, and Other Backward Classes are less likely to avail safe delivery services. Additionally, adolescent women from the southern region utilizing the highest maternal healthcare services than the other regions.

**Conclusions:**

The present study documents several socioeconomic and cultural factors affecting the utilization of maternal healthcare services among rural adolescent women in India. The ongoing healthcare programs should start targeting household with married adolescent women belonging to poor and specific sub-groups of the population in rural areas to address the unmet need for maternal healthcare service utilization.

## Introduction

Maternal healthcare remains a major challenge to the global public health system, especially in developing countries [1]. In India, considerable attention has been paid to estimates of maternal mortality, but mere has been reserved to the issue of adolescents pregnancies requires paramount attention [2]. Despite substantial improvement in maternal health indicators in India, the proportion of adolescent deaths (9%) due to pregnancy or during child birth to total maternal mortality is unacceptably high [3]. Studies have highlighted the relationships between early childbearing and adverse health outcomes potentially causing death among women in the 15–19 age groups [4], [5]. Acknowledging the importance of the issue, the United Nations focused on improving maternal health in the Millennium Development Goals to reduce Maternal Mortality Ratio (MMR) by 75% percent during 1990–2015 [6]. Additionally, adolescent pregnancies have been consistently associated with increased risk of adverse health outcomes, low birth weight, premature deliveries, high neonatal and post neonatal as well as infant morbidity and mortality [7].

The theoretical framework represented by Thaddeus and Maine (1994) referred to socioeconomic/cultural factors (women's status in household and society, educational and economic status of women etc.), accessibility to facility (distance, transportation etc.) and availability of quality of care (availability of staff and equipment in health facility centre) as the crucial factors behind maternal morbidity and mortality [8]. However, marriage at a very young age is the major reason for early pregnancy in India [7]. Studies have found that adolescents often lack experience, tend to be psychologically as well as emotionally less mature, all of which lead to poor maternal health outcome [9]. Some other factors such as education, economic status, healthcare programs and high cost of healthcare services have an impact on maternal healthcare utilization [10], [11], [12], [13], [14], [15], [16], [17]. A number of studies have discussed both accessibility and availability as determinants of health service utilization [18], [19], [20], [21].

The perspective of maternal healthcare for adolescent mothers is crucial because early sexual activity and childbearing accelerates the risk of maternal as well as child morbidity or/and mortality. These phenomena are applicable for both developed countries like the United States [22] and developing countries like India, Malaysia, Vietnam, Egypt, and South Sudan [15], [23], [24], [25], [26], [27], [28]. Recent statistics reveal that about 30%–70% of young women (aged 20–24 years) in India, Bangladesh and Nepal is married before reaching the age of 18 years [29]. A study highlighted the difference between older mothers and adolescent mothers who have high maternal and child mortality because the latter are not exposed to education due to early age at marriage, have lower contraceptive use, and more unplanned and unwanted pregnancies [7]. Adolescent childbearing has an adverse impact on three dimensions of the health of adolescent mothers as well as their infants at the individual, economic and at societal levels [5].

Adolescent mothers are more likely to have severe delivery complications resulting in high morbidity as well as mortality. There is a serious dearth of empirical research in India on the utilization of maternal healthcare services in rural settings by adolescents in the age group 15–19 years. Some studies [30], [31], [32] have focused on the rural-urban differential in healthcare utilization and found that women in rural areas have lower levels of healthcare utilization than their urban counterparts. In 2005, the Government of India launched the National Rural Health Mission (NRHM) for the improvement of the health system performance and health status of people in rural areas [33]. The NRHM was launched countrywide, with special focus on 18 states - Arunachal Pradesh, Assam, Bihar, Chhattisgarh, Himachal Pradesh, Jharkhand, Jammu and Kashmir, Manipur, Mizoram, Meghalaya, Madhya Pradesh, Nagaland, Orissa, Rajasthan, Sikkim, Tripura, Uttaranchal and Uttar Pradesh – with either weak public health indicators or poor public health infrastructure. The main objective of NRHM was to reduce child and maternal mortality by providing universal access to equitable, affordable, accountable and effective primary healthcare services to women in rural areas [34]. Additionally, *Janani Suraksha Yojana (JSY)*, a conditional cash transfer scheme was launched under the broad umbrella of the NRHM to promote institutional delivery among women in rural areas. It is expected that the promotion of institutional delivery will reduce maternal and neonatal mortality among pregnant women in rural areas with special attention to women having low socioeconomic status [35].

This paper attempts to assess the factors associated with selected maternal healthcare indicators with reference to adolescent mothers in the age group 15–19 years living in rural India. Three key indicators in healthcare are measured: adolescent women receiving full antenatal care, those who had safe delivery and adolescent women who received postnatal care within 42 days of delivery. It is hoped that the findings will help ongoing program and policy efforts to identify the key factors in the provision and utilization of maternal healthcare for rural adolescent women.

## Materials and Methods

### Data

The present study utilizes data from the third round of the Indian counterpart of Demographic and Health Survey, popularly known as National Family Health Survey carried out during 2005–06 [36]. The National Family Health Survey (NFHS) is a large-scale, multi-round survey conducted in a representative sample of households throughout India. The survey covers a representative sample of 1,24,385 women in the age group 15–49 years from all 29 states. The survey provides essential state and national level data to monitor health and family welfare programs and policies implemented by the Ministry of Health and Family Welfare and other ministries and agencies. In addition to the indicators covered in NFHS 1 and 2, the third round of NFHS (NFHS-3) provides information on several new and emerging issues such as perinatal mortality, adolescent reproductive health, high risk sexual behaviour, family life education, safe injections and knowledge about tuberculosis. The third wave of NFHS conducted in 2005–06 was the outcome of the collaborative efforts of many organizations such as the United States Agency for International Development (USAID), the United Kingdom Department for International Development (DFID), the Bill and Melinda Gates Foundation, UNICEF, UNFPA, and the Government of India. However, the technical assistance was provided by Macro International, Maryland, USA. About eighteen research organizations were involved in the fieldwork of NFHS-3, of which thirteen were private sector research organizations and five were Population Research Centres (PRCs) established by the Government of India in various states. Each research organization had the responsibility of collecting the data in one or more states. The International Institute for Population Sciences (IIPS), Mumbai, India was designated as the nodal agency for conducting, monitoring and disseminating the results of third round of National Family Health Survey.

### Sampling plan

A multistage stratified sampling method was used to create a sample, representing individuals from all 29 Indian states [36], [37]. The urban and rural samples within each state were drawn separately and a uniform sample design was adopted in all the states. In each state, the rural sample was selected in two stages, with the selection of Primary Sampling Units (PSUs), which are villages, with probability proportional to population size (PPS) at the first stage, followed by the random selection of households within each PSU in the second stage. In rural areas, a list of villages from the 2001 Census served as the sampling frame and it was stratified by a number of variables. The first level of stratification was geographic, with districts being subdivided into contiguous regions. Within each of these regions, villages were further stratified using selected variables from the following list: village size, percentage of males working in the non-agricultural sector, percentage of the population belonging to scheduled castes or scheduled tribes, and female literacy. In every state, mapping and household listing operations were carried out in each sample area. The listing provided the necessary frame for selecting households at the second stage. The households to be interviewed were selected with equal probability from the household list in each area using systematic random sampling. In NFHS-3, the data were collected using different interview schedules, which include household schedule, eligible women's schedule and men's schedule. The household response rate for rural areas in NFHS-3 was 99% and the individual response rate was 96% [37].

### Study Population and Sample Size

The present study examines the utilization of maternal healthcare services among married adolescent mothers in rural areas. The term, ‘adolescent mother’ refers only to ever married women who have had the experience of childbirth in their teens (15–19) during the five years preceding the survey date. In NFHS-3, out of all ever-married women interviewed 23,955 were in the age group 15–19 years. The data recorded 56,438 births that occurred in the five years preceding the survey. Among the women interviewed, 5,253 were found to have had experience of childbirth in their adolescence (aged 15–19 years) during the five years preceding the survey date. However the present study focused on rural areas, where about 80% (3,599) of the total adolescent women (5,253) had experience of childbirth in their adolescence. Therefore, the present study takes into consideration the responses of those adolescent mothers who had experience of childbirth in their adolescence (aged 15–19 years) during the five years preceding the survey date and residing in rural areas.

### Outcome Measurements

The study measures three outcome variables namely, full antenatal care, safe delivery and postnatal care as the indicators of maternal healthcare utilization. The three selected indicators of maternal healthcare utilization and their components are considered on the basis of guidelines developed by the Ministry of Health and Family Welfare, Government of India and the World Health Organization [38], [39]. Full antenatal care includes those mothers who had a minimum of three antenatal visits, at least two tetanus toxoid injections during the pregnancy, or received one tetanus toxoid injection during the pregnancy and at least one in the three years prior to the pregnancy, and received iron and folic acid tablets for 90 days or more [39], [40]. The provision of all components of antenatal care to pregnant women is an integral part of the Reproductive and Child Health Program in India [39]. Delivery conducted either in a medical institution or home deliveries assisted by doctor/nurse/Lady Health Visitor (LHV)/Auxiliary Nurse Midwife (ANM)/other health professionals are termed as safe delivery [37]. The study considered postnatal care check-up within 42 days after child birth as a potential maternal healthcare service indicator [41], [42].

### Defining Predictor Variables

Socioeconomic and demographic predictors such as age of the woman at birth, women's education, husband's education, religion, social group, women's autonomy, mass media exposure, wealth quintile, family structure, birth order and interval, status of the child, visit of health provider and region of residence were included as predictor variables in the study. Mother's age at birth was categorized into <18 years and 18–19 years of age. The educational level of the women and their husbands was defined using years of schooling and they were grouped into illiterate, literate but below primary, primary but below middle school, middle but below high school, and high school and above. The religion of the mother was categorized as Hindu, Muslim, and others (Sikh, Christians, Buddhist and others). Identification of the social group was based on the women's self-reporting as others, Scheduled Castes (SCs), Scheduled Tribes (STs) and Other Backward Classes (OBCs). The Central Government of India classifies certain castes/tribes based on their historical disadvantage in social and economic positions [43]. The list is dynamic (castes and communities can be added or removed) and has changed over time depending on social and economic factors [44]. Articles 340, 341 and 342 and subsequent amendments of the Indian Constitution identified lower-caste groups and classified them as the “Other Backward Classes”, “Scheduled Castes” and “Scheduled Tribes” respectively [36], [45]. The Constitution (Scheduled Castes) Order, 1950 lists 1,108 castes across 25 states while the Constitution (Scheduled Tribes) Order, 1950 lists 744 tribes across 22 states of India [46]. The First Backward Classes Commission was set up by a presidential order in 1953 and the Commission submitted its report in 1955. It prepared a list of 2,399 “backward classes” or communities for the entire country, of which 837 were classified as the most backward. Several policies and programs were introduced to facilitate the use of services and resources across all social groups at equal measure and people belonging to these groups are now offered equal access to education, employment, subsidized food, healthcare, legal aid, financial loans and so on [47], [48].

Women's autonomy was computed by taking three dimensions into account, namely, decision-making authority, women's mobility (freedom to visit places unescorted) and access to economic resources [49]. The NFHS-3 data provided information about all the three dimensions, and gave scope to construct an index to assess women's autonomy. The autonomy index was computed from the information obtained with regard to decision to go to a health facility, involvement in major and daily household purchases, decision to visit family or relatives and decision to spend the husband's money; being allowed to go out, to the market, and outside the village/community. Economic security was assessed by two indicators, namely, access to money for own use and own bank account. A higher weight was allocated if the women were involved in decision-making, if they did not require permission to go out and had economic security. The women's autonomy index has been categorized as low and high autonomy. Mass media exposure has been assessed by considering how often the respondents read the newspaper, listen to the radio and watch television or cinema. Similarly, a relative index of household wealth was also calculated from the standard set of assets owned by the household, which included ownership of consumer items and dwelling characteristics. Individuals were ranked on the basis of their household scores and divided into different quintiles, each representing 20 percent of the score, between 1 (poorest) and 5 (wealthiest) [36]. The family structure of the women's household is coded into two categories, namely, nuclear households and joint households. A nuclear household is defined as one that consists of parents and their unmarried children. The birth order of children of adolescent women and the interval between the child births were grouped as first birth order, birth order 2/3 and interval < = 24 and birth order 2/3 and interval >24.

The survey provides the information on recent contact of respondents with different health workers. The term ‘health worker visit’ encompasses a visit by any health worker namely, Auxiliary Nurse-Midwives (ANM), Lady Health Visitors (LHVs), *Anganwadi* Workers (AWW), Accredited Social Health Activists (ASHA), Multipurpose Workers (MPW), and other community health workers. Since the regional variation in the utilization of maternal healthcare was evident [36], attention was paid to adjust the estimates for region of residence. For this purpose, India was divided into six regions based on geographical location and cultural settings. The six regions consist of North (Jammu and Kashmir, Himachal Pradesh, Punjab, Haryana, Rajasthan, Delhi and Uttaranchal), Central (Uttar Pradesh, Madhya Pradesh and Chhattisgarh), East (Bihar, Jharkhand, West Bengal and Orissa), North-East (Arunachal Pradesh, Assam, Manipur, Meghalaya, Mizoram, Nagaland, Sikkim and Tripura), West (Gujarat, Maharashtra and Goa), and South (Andhra Pradesh, Karnataka, Kerala and Tamil Nadu).

### Analytical Approach

To identify the factors associated with maternal healthcare utilization among adolescent women, bi-variate and multivariate analyses were performed. Bi-variate analyses were performed to examine the nature of association between utilization of maternal healthcare services by selected socioeconomic and demographic background characteristics. However, binary logistic regression was applied to investigate which factors best explain and predict the utilization of all three maternal health outcomes. Instead of the linear probability model, logistic regression function is preferable to fit some kind of sigmoid curve when the response variable is dichotomous (i.e., binary or 0–1) and that reasonably portrays the reality about outcome events. The binary response (y, full antenatal care received or not; undergone safe delivery care or not; received postnatal care or not) for each individual was related to a set of categorical predictors, X, and a fixed effect by a logit link function as following.
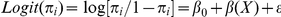



The probability of an individual who had received full antenatal care or undergone safe delivery care or received postnatal care is π_i_. The parameter β_0_ estimates the log odds of full antenatal care or safe delivery care or postnatal care for the reference group, and the parameter β estimates with maximum likelihood, the differential log odds of full antenatal or safe delivery or postnatal care are associated with the predictor X, as compared to the reference group. It is worth mentioning, ε represents the error term in the model. In the bi-variate analysis, using the Chi-square test, significant variables were identified and those were included in the binary logistic regression model. The results of logistic regression are presented by estimated odds-ratio with 95% Confidence Interval (CI). The whole analysis was performed using SPSS version 15.0 [50].

### Ethical Statement

This study uses the National Family Health Survey dataset which is available to the researchers upon request at <www.measuredhs.com/data/dataset/India_Standard-DHS_2006.cfm?flag=0>. The survey was approved by the International Institute for Population Sciences (IIPS) ethical review board in India and the institutional review boards of the funding agencies and the technical assistance agencies. Data collection procedures were also monitored and approved by the ORC Macro institutional review board. All individuals selected in the National Family Health Survey were provided with informed voluntary as well as written consent. Each individual's approval was sought, and only then was the interview conducted.

## Results

Table 1 shows differences in some key indicators between rural women who had delivered their last child in the age group 15–19 years and women in the age group 20 and above. The mean age at marriage was 15 years among women who gave birth in the age group 15–19 years, while it was 17 years among women who gave birth in the age group 20 years and above. Among rural adolescents who had given birth to their last child in the age group 15–19 years, the mean age at first birth was 16.8, while the corresponding figure was 19.5 for the women who gave birth in the age group 20 years and above. Among rural adolescent women who gave birth in the age group 15–19 years, 35% had no mass media exposure and 33% were from the poorest wealth quintile. More than half of the children born to the rural adolescent women who gave birth in the age group 15–19 years were stunted and about 15% suffered from diarrhea. The current contraceptive use among rural adolescent women who had given birth to their last child during adolescence was substantially low and about 15% experienced heavy vaginal bleeding after delivery.

**Table 1:** Comparison of selected characteristics of married women by age at last birth in five years preceding the survey, NFHS 2005–06, India.

Selected indicators	15–19^a^	20 and above^a^	Total^b^
Mean age at marriage	15.1	17.1	17.3
Mean age at first birth	16.8	19.5	19.4
Mean highest years of schooling	3.4	3.9	4.2
% of partners with high school education and above	22.3	26.8	25.0
% with no mass media exposure	35.0	38.3	30.4
% belonging to the poorest wealth quintile	32.8	28.2	31.4
% children stunting	52.4	47.0	47.6
% children suffering from diarrhea	14.5	10.5	11.0
% currently not using any contraceptive	71.3	56.0	58.8
% experienced massive vaginal bleeding after birth	15.0	11.0	13.0
**n**	**3599**	**18724**	**36850**

Note:

a Includes rural samples only.

b Includes both urban and rural samples.

### Profile of the Respondents

Table 2 represents the weighted percentage distribution of the adolescents, who had delivered the last child during the five years preceding the survey by select background characteristics. Majority of the adolescent women (62%) had given birth before 18 years of age. About 49% of adolescent women were illiterate and majority of them belonged to the Hindu religion. The social group wise distribution shows that most of the adolescent mothers (42%) were from Other Backward Classes (OBCs). More than three-fourths of the adolescent women in the rural sample reportedly had low autonomy and 35% had no mass media exposure. About 33% and 27% adolescent women in the study were from the poorest and poorer wealth quintiles respectively. In the rural areas, about 69% of adolescent women reported that they were living in the joint family. Nearly 37% of rural adolescent women, who had given birth during the five years prior to the survey reported about the health provider's visit and more than one-fifth of them belonged to the eastern and central regions of India.

**Table 2:** Percentage distribution of women who had at least one live birth in their adolescence (15–19) during the last five years preceding the survey by background characteristics, NFHS-3 (2005–06), rural India.

Background characteristics	%	n
**Maternal age**		
<18	61.9	2180
18–19	38.1	1419
**Women's education**		
Illiterate	48.5	1612
Literate but below primary	10.1	395
Primary but below middle	21.5	787
Middle but below high school	12.5	527
High school and above	7.4	278
**Husband's education**		
Illiterate	30.9	1057
literate but below primary	8.7	333
Primary but below middle	18.9	702
Middle but below high school	19.2	726
High school and above	22.3	781
**Religion**		
Hindu	82.5	2750
Muslim	14.5	501
Others	3.0	348
**Social group**		
Others	20.9	747
Scheduled Castes (SCs)	24.1	741
Scheduled Tribes (STs)	12.6	671
Other Backward Classes (OBCs)	42.3	1271
**Autonomy**		
Low	79.4	2738
High	20.6	859
**Mass media exposure**		
No exposure	35.0	1139
Any exposure	65.0	2460
**Wealth quintile**		
Poorest	32.8	974
Poorer	27.4	1071
Middle	24.0	915
Richer	12.4	482
Richest	3.4	157
**Family structure**		
Nuclear	31.1	1012
Joint	68.9	2180
**Birth order and interval**		
Birth order 1	63.0	2303
Birth order-2/3 and interval < = 24	18.0	640
Birth order-2/3 and interval >24	19.0	656
**Status of the child**		
Wanted	85.5	3029
Unwanted	14.5	567
**Visited by the health providers**		
No	63.1	2348
Yes	36.9	1251
**Region**		
South	16.4	548
North	9.2	481
Central	25.2	803
East	35.1	881
Northeast	4.4	614
West	9.8	272
**Total number of respondents**		**3599**

Note: All ‘n’ are unweighted. Total may not be equal due to some missing cases.

### Differentials in the Utilization of Maternal Healthcare Services

To identify the factors associated with the utilization of maternal healthcare services, namely, full antenatal care, safe delivery and postnatal care, we examined the bi-variate differential of the selected socioeconomic and demographic characteristics. Table 3 shows the weighted percentage of women who utilized maternal healthcare services by selected background characteristics. Overall, 14% of the rural adolescent women received full antenatal care, 46% utilized safe delivery care and 35% had postnatal care check-ups. The rate of full antenatal care (7%) and postnatal utilization (24%) was very low among uneducated women. Similarly, safe delivery care use was 31% among women with no formal education, and the same was found to be high at 83% for those with high school education and above. Utilization of all three services was observed to be higher among those women whose husbands had high school education and above. About one in five and three in five women whose husbands had high school education and above, had utilized full antenatal care services and had safe deliveries respectively. Utilization of postnatal care was 43% among women whose husbands had high school education and above, and it was found to be low for those, whose husbands were uneducated (28%). Nearly half of the adolescent women from other religions utilized safe delivery care, while the corresponding figure for Muslim women was reported to be 37%. Similarly, postnatal care was utilized more by women from other religious groups (42%), followed by Hindu (35%) and Muslim (30%) women. The utilization of full antenatal care (10%), safe delivery (33%) and postnatal care (31%) was lowest among women from Scheduled Tribes (ST). It was also found that for women who had reported high autonomy, the utilization of safe delivery and postnatal care was 49% and 38% respectively. Women who had any exposure to mass media utilized 17% of full antenatal care; however the corresponding figure was observed to be 8% among women who had no mass media exposure. Similarly, safe delivery and postnatal care were 53% and 40% respectively among rural adolescent ever married women who had any exposure of mass media.

**Table 3:** Percentage of women who had at least one live birth in their adolescence (15–19) during the last five years preceding the survey by usage pattern of maternity care services by background characteristics, NFHS-3 (2005–06), rural India.

Background characteristics	Full antenatal care	Safe delivery	Postnatal care
**Maternal age**	(4.790)**	(0.446)ns	(1.110)ns
<18	13.0	45.3	33.9
18–19	15.1	46.2	35.3
**Women's education**	(259.469)***	(549.670)***	(316.619)***
Illiterate	6.9	31.3	24.0
Literate but below primary	16.7	48.1	36.3
Primary but below middle	18.6	54.7	41.1
Middle but below high school	19.2	61.8	46.9
High school and above	31.8	82.5	60.3
**Husband's education**	(76.994)***	(250.827)***	(78.635)***
Illiterate	8.3	32.8	27.5
Literate but below primary	12.3	38.1	31.8
Primary but below middle	15.4	45.9	37.1
Middle but below high school	15.5	51.6	34.7
High school and above	19.1	60.9	42.8
**Religion**	(3.285)ns	(29.492)***	(13.712)***
Hindu	13.5	47.1	35.1
Muslim	14.3	36.8	29.5
Others	18.3	48.2	42.3
**Social group**	(32.821)***	(114.951)***	(65.487)***
Others	17.7	56.7	45.3
Scheduled Castes (SCs)	10.8	40.1	31.4
Scheduled Tribes (STs)	9.9	33.3	31.3
Other Backward Classes (OBCs)	14.6	48.3	33.1
**Autonomy**	(0.821)ns	(7.278)***	(7.558)***
Low	13.5	44.7	33.6
High	14.6	49.2	38.0
**Mass media exposure**	(84.544)***	(219.472)***	(151.418)***
No exposure	7.9	32.0	23.7
Any exposure	16.9	53.0	40.3
**Wealth quintile**	(224.383)***	(497.739)***	(306.121)***
Poorest	7.0	29.2	23.1
Poorer	10.5	41.1	29.8
Middle	17.5	54.4	41.4
Richer	25.3	67.7	50.7
Richest	32.6	88.1	67.6
**Family structure**	(0.145)ns	(24.592)***	(3.613)*
Nuclear	13.2	39.9	32.0
Joint	13.6	47.6	34.8
**Birth order and interval**	(16.078)***	(200.393)***	(41.750)***
Birth order 1	15.1	52.6	37.4
Birth order-2/3 and interval < = 24	10.4	38.6	32.8
Birth order-2/3 and interval >24	12.4	29.2	26.7
**Status of the child**	(1.834)ns	(0.184)ns	(0.013)ns
Wanted	13.5	45.8	34.5
Unwanted	15.3	44.9	34.7
**Visited health provider**	(15.942)***	(0.158)ns	(19.140)***
No	12.3	45.4	32.3
Yes	16.2	46.0	38.2
**Region**	(337.076)***	(492.363)***	(811.924)***
South	32.0	73.7	70.4
North	11.6	50.0	37.3
Central	5.8	33.1	18.2
East	11.8	37.9	26.5
Northeast	9.3	30.8	16.9
West	14.5	61.1	49.8
**Total**	**13.8**	**45.6**	**34.5**

Note: Figures in parentheses are the Chi-square statistics; χ^2^ test applied for each variable.

Level of significance:

* p<0.10;

** p<0.05;

*** p<0.01. ns: not significant.

The utilization of all three maternal healthcare services was observed to increase with the increase in wealth quintile. For instance, only 7% of rural adolescent mothers belonging to the poorest wealth quintile received full antenatal care, while this proportion was found to be 33% among adolescents from the richest wealth quintile. A similar pattern was observed where 29% and 23% of the women belonging to the poorest wealth quintile utilized safe delivery and postnatal care respectively, compared to 88% and 68% from the richest wealth quintile. Adolescent women residing in joint families utilized more safe delivery care (48%). The utilization of full antenatal care (15%), safe delivery (53%) and postnatal care (37%) was higher among those women with first order child birth than with those who had had previous experiences of childbirth. The rates of full antenatal care (16%) and postnatal care (38%) were observed to be high among women whom health providers had visited during pregnancy. Adolescent women from the South region were found to be utilizing maternal healthcare services more than the women from other regions. However, the lowest utilization of full antenatal care (6%) was observed in the Central region. Safe delivery (31%) and postnatal care (17%) was least utilized by adolescent women belonging to the Northeast region.

### Determinants of Full Antenatal Care Utilization

Multivariate results for full antenatal care utilization presented in Table 4 reiterate that some important factors such as women's education, husband's education, economic status, birth order and interval, health provider's visit and region of residence were found to be significant determinants of the utilization of antenatal care services among rural adolescent women. Women with middle and higher education were two (CI = 1.580–2.851) and nearly three times (CI = 2.033–3.991) more likely to utilize full antenatal care than uneducated women. Moreover, the odds of receiving full antenatal care among women who had formal education but below primary level (OR = 2.003, CI = 1.483–2.707) were high compared to uneducated women. Similarly, women whose husbands had high school education and above, were more likely to utilized full antenatal care compared to women whose husbands had no formal education (OR = 1.354, CI = 1.015–1.807). The wealth quintile showed a significant positive effect on the utilization of full antenatal care among rural adolescent women. Women from the richer and richest wealth quintiles were nearly two (CI = 1.326–2.518) and two and half times (CI = 1.628–4.094) more likely to use full antenatal care respectively compared to women from the poorest wealth quintile. Women with two or three birth orders children and < = 24 months of birth interval were less likely to utilize full antenatal care than women who had experienced childbirth for the first time (OR = 0.634, CI = 0.494–0.814). A visit by health providers led to a significant increase in the utilization of full antenatal care. The odds of receiving full antenatal care was higher (OR = 1.430, CI = 1.200–1.703) among women whom the health provider visited compared to those women whom the health provider had not visited. Another significant finding is the regional variation in the utilization of antenatal care. Full antenatal care utilization was found to be less likely in all other regions of India, compared to the Southern region. The lowest odds of full antenatal care utilization among adolescent women were evident in the Central region (OR = 0.147, CI = 0.110–0.195) followed by the North region (OR = 0.250, CI = 0.177–0.352) and Northeast region (OR = 0.259, CI = 0.156–0.429) with reference to South region.

**Table 4:** Binary Logistic Regression Model showing Odds Ratio and 95% Confidence Interval (CI) for receiving full antenatal care among women who had at least one live birth in their adolescence (15–19) during the last five years preceding the survey, NFHS-3 (2005–06), rural India.

Covariates	Odds ratio	95% CI
**Maternal age**		
<18®	1.000	
18–19	1.069ns	0.893–1.279
**Women's education**		
Illiterate®	1.000	
Literate but below primary	2.003***	1.483–2.707
Primary but below middle	2.152***	1.686–2.748
Middle but below high school	2.123***	1.580–2.851
High school and above	2.848***	2.033–3.991
**Husband's education**		
Illiterate®	1.000	
Literate but below primary	1.419*	0.994–2.024
Primary but below middle	1.409**	1.070–1.856
Middle but below high school	1.436*	1.080–1.909
High school and above	1.354**	1.015–1.807
**Social group**		
Others®	1.000	
Scheduled Castes (SCs)	0.827ns	0.637–1.075
Scheduled Tribes (STs)	0.872ns	0.627–1.214
Other Backward Classes (OBCs)	0.858ns	0.690–1.068
**Mass media exposure**		
No exposure®	1.000	
Any exposure	1.092ns	0.872–1.366
**Wealth quintile**		
Poorest®	1.000	
Poorer	1.139ns	0.871–1.491
Middle	1.447***	1.098–1.907
Richer	1.827***	1.326–2.518
Richest	2.582***	1.628–4.094
**Birth order and interval**		
Birth order 1®	1.000	
Birth order-2/3 and interval < = 24	0.634***	0.494–0.814
Birth order-2/3 and interval >24	0.855ns	0.674–1.083
**Visited health providers**		
No®	1.000	
Yes	1.430***	1.200–1.703
**Region**		
South®	1.000	
North	0.250***	0.177–0.352
Central	0.147***	0.110–0.195
East	0.318***	0.254–0.399
Northeast	0.259***	0.156–0.429
West	0.267***	0.197–0.363

Note: ®:Reference Category.

Level of significance:

* p<0.10;

** p<0.05;

*** p<0.01. ns: not significant.

### Determinants of Safe Delivery Care Utilization

Results of the multivariate analysis for safe delivery care are presented in Table 5. Findings show that women's education, religion, social group, women's autonomy, mass media exposure, economic status, birth order and interval, and region were found to be statistically significant determinants in the utilization of safe delivery.

**Table 5:** Binary Logistic Regression Model showing Odds Ratio and 95% Confidence Interval (CI) for receiving safe delivery among women who had at least one live birth in their adolescence (15–19) during the last five years preceding the survey, NFHS-3 (2005–06), rural India.

Covariates	Odds ratio	95% CI
**Women's education**		
Illiterate®	1.000	
Literate but below primary	1.747***	1.384–2.204
Primary but below middle	2.010***	1.674–2.414
Middle but below high school	2.123***	1.682–2.679
High school and above	3.876***	2.715–5.533
**Husband's education**		
Illiterate®	1.000	
Literate but below primary	0.923ns	0.708–1.204
Primary but below middle	1.239*	1.013–1.515
Middle but below high school	1.409**	1.142–1.738
High school and above	1.210*	0.966–1.514
**Religion**		
Hindu®	1.000	
Muslim	0.652***	0.513–0.828
Others	0.746ns	0.498–1.119
**Social group**		
Others®	1.000	
Scheduled Castes (SCs)	0.613***	0.491–0.765
Scheduled Tribes (STs)	0.496***	0.382–0.642
Other Backward Classes (OBCs)	0.714***	0.587–0.869
**Autonomy**		
Low®	1.000	
High	1.174*	0.986–1.397
**Mass media exposure**		
No exposure®	1.000	
Any exposure	1.289***	1.105–1.503
**Wealth quintile**		
Poorest®	1.000	
Poorer	1.206**	1.008–1.444
Middle	1.376***	1.125–1.685
Richer	1.680***	1.286–2.196
Richest	3.610***	1.091–5.182
**Family structure**		
Nuclear®	1.000	
Joint	0.967ns	0.824–1.134
**Birth order and interval**		
Birth order 1®	1.000	
Birth order-2/3 and interval < = 24	0.487***	0.407–0.585
Birth order-2/3 and interval >24	0.321***	0.265–0.388
**Region**		
South®	1.000	
North	0.274***	0.204–0.367
Central	0.154***	0.122–0.195
East	0.218***	0.175–0.272
Northeast	0.104***	0.069–0.156
West	0.371***	0.281–0.490

Note: ®: Reference Category.

Level of significance:

* p<0.10;

** p<0.05;

*** p<0.01. ns: not significant.

The utilization of safe delivery care increases with the level of women's education. Compared to uneducated rural adolescent women, those who had completed high school education and above, were more likely to utilize safe delivery care (OR = 3.876, CI = 2.715–5.533). The likelihood of using safe delivery was observed to be low among women belonging to the Muslim religion (OR = 0.652, CI = 0.513–0.828) compared to women belonging to the Hindu religion. The probability of utilizing safe delivery was found to be less likely among STs (OR = 0.496, CI = 0.382–0.642), SCs (OR = 0.613, CI = 0.491–0.765) and OBCs (OR = 0.714, CI = 0.587–0.869) than among women from the other social groups. Women who had exposure to mass media were more likely to utilize safe delivery care than women who did not have any mass media exposure (OR = 1.289, CI = 1.105–1.503). Economic status was also found to be an important significant determinant in the utilization of safe delivery care. Adolescent women from the richer and richest wealth quintiles were 1.7 (CI = 1.286–2.196) and 3.6 times (CI = 1.091–5.182) more likely to use safe delivery care respectively compared to those from the poorest wealth quintiles. The probability of safe delivery care was found to be less likely among women who had birth order 2/3 and birth interval >24 months than among women who experienced their first childbirth (OR = 0.321, CI = 0.265–0.388). The regional variation shows that compared to the south region, the odds of utilizing safe delivery care were observed to be the lowest in the northeast region (OR = 0.104, CI = 0.069–0.156), followed by the central (OR = 0.154, CI = 0.122–0.195) and east regions (OR = 0.218, CI = 0.175–0.272).

### Determinants of Postnatal Care Utilization

Table 6 demonstrates the results of the multivariate analyses on the use of postnatal care by rural adolescent women in India. The findings show women's education, social group, mass media exposure, wealth quintile, birth order and interval, health provider's visit and region as significant factors affecting postnatal care utilization. The odds of receiving postnatal care by women with primary (OR = 1.588, CI = 1.309–1.927) and middle education (OR = 1.912, CI = 1.501–2.434) were more compared to uneducated women. Also, women who were literate, but who had below primary level education were more likely to utilize postnatal care compared to uneducated women (OR = 1.417, CI = 1.112–1.806). Postnatal care utilization was found to be less likely among SCs (OR = 0.693, CI = 0.555–0.865), STs (OR = 0.706, CI = 0.545–0.915) and OBCs (OR = 0.584, CI = 0.481–0.709) than among other social groups. The likelihood of utilizing postnatal care was found to be nearly three times higher (CI = 1.729–4.347) among women from the richest wealth quintile than among those from the poorest wealth quintile.

**Table 6:** Binary Logistic Regression Model showing Odds Ratio and 95% Confidence Interval (CI) for receiving postnatal care among women who had at least one live birth in their adolescence (15–19) during the last five years preceding the survey, NFHS-3 (2005–06), rural India.

Covariates	Odds ratio	95% CI
**Women's education**		
Illiterate®	1.000	
Literate but below primary	1.417***	1.112–1.806
Primary but below middle	1.588***	1.309–1.927
Middle but below high school	1.912***	1.501–2.434
High school and above	1.917***	1.399–2.627
**Husband's education**		
Illiterate®	1.000	
Literate but below primary	1.059ns	0.804–1.394
Primary but below middle	1.286*	1.042–1.587
Middle but below high school	1.070ns	0.855–1.339
High school and above	1.026ns	0.811–1.297
**Religion**		
Hindu®	1.000	
Muslim	0.877ns	0.686–1.121
Others	0.918ns	0.618–1.365
**Social group**		
Others®	1.000	
Scheduled Castes (SCs)	0.693***	0.555–0.865
Scheduled Tribes (STs)	0.706***	0.545–0.915
Other Backward Classes (OBCs)	0.584***	0.481–0.709
**Autonomy**		
Low®	1.000	
High	1.106ns	0.926–1.320
**Mass media exposure**		
No exposure®	1.000	
Any exposure	1.230**	1.044–1.449
**Wealth quintile**		
Poorest®	1.000	
Poorer	1.021ns	0.841–1.239
Middle	1.183ns	0.956–1.464
Richer	1.360**	1.038–1.783
Richest	2.741***	1.729–4.347
**Family structure**		
Nuclear®	1.000	
Joint	0.922ns	0.783–1.086
**Birth order and interval**		
Birth order 1®	1.000	
Birth order-2/3 and interval < = 24	0.680***	0.563–0.821
Birth order-2/3 and interval >24	0.547***	0.450–0.664
**Visited health provider**		
No®	1.000	
Yes	1.455***	1.259–1.681
**Region**		
South®	1.000	
North	0.219***	0.165–0.291
Central	0.089***	0.070–0.113
East	0.157***	0.127–0.193
Northeast	0.068***	0.043–0.107
West	0.309***	0.238–0.400

Note: ®: Reference Category.

Level of significance:

* p<0.10;

** p<0.05;

*** p<0.01. ns: not significant.

Effect of birth order and interval appeared to be a significant factor affecting postnatal care utilization among adolescent women. Adolescent women with the second or third birth order child utilized less postnatal care than women with the first birth order child. The odds of utilizing postnatal care were found to be low among women with second or third order births with less than < = 24 months of previous birth interval compared to women with first order births (OR = 0.680, CI = 0.563–0.821). Similarly, women with second or third order births of children and with more than 24 months of previous birth interval were also less likely to utilize postnatal care than the first order birth adolescent mothers (OR = 0.547, CI = 0.450–0.664). The odds of utilizing postnatal care were found to be more likely among women who had been recently visited by health workers compared to women who had no contact with any health workers (OR = 1.455, CI = 1.259–1.681). Region of residence again emerged as a strong factor affecting the utilization of postnatal care. Adolescent mothers from the northeast region were found to be the lowest users of postnatal care compared to women from the South region (OR = 0.068, CI = 0.043–0.107).

## Discussion

Ever since the integration of the Safe Motherhood and Child Health Program into the Reproductive and Child Health Program (RCH) in 1996, the Government of India has made several efforts to improve the maternal healthcare utilization. Moreover, because of the low age at marriage pattern and early childbearing trend, utilization of maternal care services among adolescent women has been always a central focus among policy makers. Thus, considering the distinct disadvantage over urban dwellers, the present study assesses the utilization of maternal healthcare services among rural adolescent women who had given birth in the five years preceding the survey. The study has used data from the National Family Health Survey (NFHS) conducted during 2005–06. The objective of the study is to examine the factors that significantly affect the use of maternity care services, namely, full antenatal care, safe delivery and postnatal care among married adolescents in rural India. This study has investigated the factors affecting the use of maternity care services, with the aim of improving the information available to decision-makers who are responsible for planning and administering maternal care programs.

The findings of this study show unacceptably low utilization of maternity care services among adolescent ever married women in rural India. Only about 14%, 46%, and 35% of adolescent women from rural areas received full antenatal care, safe delivery and postnatal care services respectively. A recent cross-sectional survey titled, ‘Youth in India: Situation and Needs Study’ conducted in six states (Andhra Pradesh, Bihar, Jharkhand, Maharashtra, Rajasthan, and Tamil Nadu) of India has revealed that in six selected states, nearly 47% rural married women aged 15–24 utilized safe delivery care, while it was about 78% among urban women [51]. This contrast between rural and urban areas is a powerful indication of the stark disparity that exists by place of residence in the utilization of maternal healthcare services in India. This study has also identified several other determinants that have a significant influence on the utilization of maternal healthcare services such as women's education, social group, religion, economic status, birth order and interval, health provider's visit and region of residence.

The results from this study show that maternal education exerts a significant influence on the utilization of maternal healthcare services by adolescent women, after controlling for other selected covariates. However, the effect of education is not constant across all educational levels, nor is it the same for the three different types of maternal health services. Many studies conducted in other developing countries have found that maternal education is one of the most important determinants of maternal healthcare utilization, after controlling for other factors [11], [14], [16], [52], [53], [54], [55], [56], [57]. The net effect of education on maternal healthcare services utilization among rural adolescent women is significantly interlinked with low age at marriage and early childbearing. Despite several governmental and non-governmental efforts to delay age at marriage, nearly fifty percent of Indian women in the age group 20–24 were married by 18 years, and this proportion is observed to be as high as one in two to three in five in several states [24], [36]. In rural areas particularly, adolescent women are forced to leave school after marriage in order to spend time on childrearing and other household chores [58]. There are a number of explanations for why women's education is a key determinant affecting the utilization of maternal healthcare services. Education enhances communication with the husband and other family members on health related issues [12]. It helps women develop greater confidence to make decisions regarding their health. Educated women seek out higher quality services and have a greater ability to use healthcare inputs to improve their health. This finding is consistent with that documented in previous research - educated women are more likely to be aware of the benefits of healthcare services and as a result are more likely to use healthcare services [59]. Moreover, education imparts feelings of self-worth and self-confidence which are critical in bringing about changes in health-related behavior [60].

The utilization of safe delivery care was found to be significantly lower among Muslim women than among women belonged to other religions. However, few studies observed mixed effects of religion on maternal care services utilization [61]; Hindu and Muslim women residing in urban India do not avail delivery care services equally as documented earlier [62]. A study conducted in Kerala, found that Muslim women were less likely to deliver a baby in a heath care institution than Hindu women, but the possible causes remained unexplained [12]. A recent study [63] suggested a possibility that the ‘*purdah*’, a physical segregation of the sexes, and the requirement for women to cover their bodies and conceal their form [64], may be contributing to the low utilization of safe delivery care. There has been considerable discussion about the influence of religious and cultural practices on overall health behavior independent of socioeconomic processes [65], [66], [67]. In India, the religious differentials in the socioeconomic and demographic profiles have been well documented due to cultural and regional diversity [68], [69], [70], [71] and, political interests [72], [73]. The most studied issue in public health literature in India has been on Hindu-Muslim differences in fertility and family planning [74], [75], [76]. Few studies have also explored child survival prospects across different religious groups [77]. A study in India highlighted that Muslim women tend to put the baby to the breast sooner after birth and this may perhaps explain the Muslim child survival advantage [78]. The same study observed that Muslim women gave birth outside medical facilities more often than their Hindu counterparts, thus reinforcing the puzzle. Although studies highlighted lower coverage of health services utilization by Muslim women, no clear explanations emerged. Studies on fertility in India and in developing countries could perhaps throw light on the extent of disadvantage that exists among Muslim women in maternal healthcare utilization. Also, the lower maternal healthcare utilization among adolescent Muslim women could be linked to their lower socioeconomic status [31], [69].

In 2005, a committee was appointed by the Prime Minister of India to conduct a systematic study of the social, economic and educational status of the Muslim community. The report of this commission, referred to as the Sachar Report [79], concluded that Muslims ‘exhibit deficits and deprivation in practically all dimensions of development’ and ‘the deficits are particularly salient in the areas of female schooling and economic status’. Studies from South Asian countries, including Pakistan [80], [81] and Bangladesh [82] observed that religion is an important predictor of demographic and health outcomes, independent of socioeconomic factors. Evidence from Africa reflected that the high level of maternal health service utilization among women of certain religious groups could be attributed to lifestyle and theological differences between different religions [83]. Further, certain norms and characteristics of some religious groups may encourage negative attitudes toward modern medicines and health services utilization [84]. There is a clear need for future research in this area to identify the barriers towards healthcare utilization by adolescents Muslim women in rural areas. The relevance of religious differences in maternal healthcare service utilization among adolescent women has some policy implications too. Considering the interaction that religion fosters, its inclusion in the overall maternal health educational programs can facilitate the dissemination of the relevance of such services. Also, there is the need to include religious bodies in this effort, as evidence from Uganda suggests that collaboration between religious leaders and health officials are vital for changes in health and risky behavior [85]. Also, attention needs to be drawn to the issues of divine healing as well as social support networks and interaction that religion fosters as providing an environment for perhaps the diffusion of health related ideas [84], particularly among adolescents women residing in rural settings.

Although, social group was not a significant factor affecting utilization of full antenatal care, it was a significant predictor for safe delivery and postnatal care utilization among rural adolescent women. Women from Scheduled Castes (SCs), Scheduled Tribes (STs) and Other Backward Classes (OBCs) were less likely to have safe delivery and postnatal care utilization. Despite several affirmative efforts by central and state governments, social group as a significant determinant in health services utilization still persists [86]. Previous studies have shown that fewer ‘lower castes’ women receive maternal healthcare services [12] such as antenatal care [87], [88] having a trained attendant present at birth [12], [14], [89] and contraceptive use [90] compared to women belonging to ‘upper castes’. Previous studies in India have long argued that social group could be considered broadly as a proxy for socioeconomic status and poverty [44], [91]. Moreover, Scheduled Castes and Scheduled Tribes are considered socially disadvantaged groups and such groups have a higher probability of living under adverse conditions [92]. The low utilization of maternal healthcare among certain social groups indicates that there could be lack of access to healthcare services among socially backward communities. A study conducted in rural north India [86] highlighted that majority of untrained/traditional birth attendants called *Dais* belong to the lower caste, and trained birth attendants, such as nurses or doctors belong to the upper caste. Since the delivery of maternal healthcare involves the socially prohibited physical contact between the two caste groups, upper caste healthcare providers prefer to cater to the upper caste women and lower caste women choose to seek care from the traditional birth attendants to avoid the embarrassment of caste discrimination. Unlike urban areas, SCs and STs usually live in a separate habitation in rural areas, away from the main settlement. Thus, the spatial disadvantage combined with social and economic seclusion, could be the reason for the relative underutilization of maternal healthcare services among rural adolescent women belonging to SCs, STs and sometimes even the OBCs. Dissemination of information and generating awareness about the availability of subsidized maternal health services at the government-run health facilities could improve maternity services among adolescent women of certain social groups.

The disparity in the use of maternal healthcare utilization across economic groups is also an area of concern among policy makers. Economic status was found to be a significant factor affecting the utilization of maternal healthcare services in India. Adolescent women belonging to the wealthier groups were more likely to use maternal healthcare services than poor adolescent women [93]. In India, the poor-rich gap in the utilization of maternal healthcare services has been confirmed by previous studies [21], [94]. Low coverage of maternal healthcare utilization among poor households could be due to the low priority assigned to health seeking to other basic daily living needs. Moreover, poor households do not have the resources for healthcare expenses, whereas wealthier households can spend a higher proportion of their earnings on healthcare [95], [96]. Poor young women are often found to be uneducated, unemployed, and detached/excluded from social networks; they are thus less easily reached by programs that rely on mass media for diffusion of information for existing health services utilization [97].

Health worker's visits have a significant influence on the utilization of full antenatal care and postnatal care services among rural adolescent women. Other studies in India have also focused on the positive impact of the visit of a health worker, especially during pregnancy, on the utilization of maternal healthcare services [11]. This result indicates the potential importance of a health worker's visit in providing antenatal and postnatal care. The effect of birth order appears to be consistent in the utilization of all three maternal healthcare services. Some studies have highlighted limited care during the antenatal period and delivery for the second and higher order births than for the first birth [98]. Evidence from Kerala, which ranked high in the use of maternal healthcare services as well as social development, has also shown more pronounced effect of birth order on the use of maternal healthcare services than in other states [12]. This could be because women with a first child are probably more cautious about their pregnancy and are usually more likely to have difficulties during labor and delivery than women of high parity [7], [99]. This may result in low parity women being more motivated to utilize maternity care than high parity women. Further, as the order of birth increases, women may rely more on their previous experience and knowledge. A possible explanation for the low utilization of delivery care services among women from higher order birth is that such women could have developed confidence and may believe that modern healthcare is not as necessary as they had gained experience and knowledge from previous pregnancies and births [15]. Also in the Indian cultural context, a woman goes to her mother's place for delivery, particularly for the first pregnancy. The place of delivery and delivery assistance in this case is determined primarily by parents' characteristics; it is expected that parents give the best possible care to their daughters during pregnancy and childbirth [12], [100]. For these reasons, the likelihood of utilization of maternal healthcare services could be declining with higher birth order. However, the present study proposes to address households with higher order young mothers by local health workers' visit for appropriate counseling regarding the utilization of maternal healthcare services.

In India, healthcare policy makers frequently discuss regional disparities in the utilization of healthcare services. Results from this study clearly illustrate the importance of region of residence in determining maternal healthcare service utilization among rural adolescent. Results show that compared to the southern region, adolescent women living in rural areas of other regions of the country were found to be significantly less likely to use all three maternal healthcare services. This variation in the utilization of maternal healthcare services across the different regions of India may be linked with the state of socioeconomic and demographic progress. States belonging to the central and eastern regions of India together account for 55% of the total population living below the poverty line [101]. Importantly, half of the maternal deaths during 2007–09 were contributed by these states [3]. Moreover, states in the north, central and east regions include eight Empowered Action Group (EAG) states characterized by low women's education, poor exposure to mass media and low mean age at marriage. On the contrary, most of the southern states are more economically and demographically advanced than the northern and eastern states of India [102], [103]. These states accounted comparatively low maternal deaths with a relatively higher use of maternal care services [36]. In addition, states belonging to the southern region have achieved replacement level fertility and have an improved socioeconomic status [47], [103] compared to other regions of India. However, to address the regional inequality in health service utilization, in rural areas, the Government of India had launched the National Rural Health Mission (NRHM) which focuses on the states belonging to north, central and northeast regions.

A conditional cash incentive scheme called *Janani Suraksha Yojna (JSY)* under NRHM was initiated to encourage pregnant women from rural areas to deliver in healthcare institutions. The scheme encourages pregnant women from rural areas to avail themselves of maternal healthcare services (antenatal, natal and postnatal care). A recent evaluation study on JSY in a few selected districts in the northern and central states found a significant increase in institutional delivery among women in rural areas [104]. However, states like Rajasthan in the North, Uttar Pradesh, Chhattisgarh and Madhya Pradesh in the Central region and Orissa and Jharkhand in the East require special attention as one in four women in the age group 15–19 reported childbirth, coupled with lower contraceptive prevalence rate [36].

To conclude, the present study has documented that the utilization of maternal healthcare services among rural adolescent women is far from acceptable. Low coverage of these services could lead to adverse health outcomes for both the mother and the child. Earlier reproductive health programs in India have paid limited attention to married adolescent girls as a separate category, typically grouping all married women together regardless of current age, age at marriage, and socioeconomic characteristics. The present study emphasizes on specific programs and schemes that acknowledge the need of adolescents and newlywed young mothers living in rural areas in particular. The results of this study have scope for providing the basis for a few policy implications.

First, education was found to have a significant impact on the use of maternal healthcare services. This suggests that improving educational opportunities to adolescent women may have a large impact on the utilization of maternal healthcare services. Promoting higher education for the girl child has been identified as the most effective way to address low coverage of maternal healthcare utilization. In view of this, government policies and programs to improve higher educational opportunities for rural adolescent women need to be reinforced. A recent study indicated that schooling was far from universal among young women in the country; one in four young women had never been to school according to a recently conducted ‘Youth in India: Situations and Needs’ study [51]. Moreover, married young women and those in rural areas were far more disadvantaged than the unmarried and those in urban areas [51]. In majority of the developing countries, with the emphasis on ‘cost-sharing’ in education, many parents are likely to educate their sons at the expense of their daughters; this is an issue that has long been derailing the progress in female education [23]. Although India has registered impressive gains in educational attainment during the last few decades [105], educational reforms still need to focus extensively on reducing costs on education and providing financial support for the girl child residing in rural areas. Second, women with higher order birth were less likely to use maternity care services and this suggests that birth order should be used as a condition for targeting educational and awareness campaigns on the benefits of ongoing safe motherhood programs in rural areas. Third, existing government policies and programs should target households with married adolescent women belonging to poor and specific sub-groups (like religion, social group) of the population in rural areas to address the unmet need for maternal healthcare service utilization. In order to reduce maternal illness and death through proper and timely maternity services utilization, more investment and interventions among the poorest and specific sub-groups of girls are needed, as the same girls are being forced in to child marriage and harmful traditional practices.

There is need for building awareness on the issue of early marriage and adverse effects of early pregnancy at the family and societal levels. Recent studies have emphasized the need to work within existing community structures and attempt to bring awareness to communities about how child marriage compromises opportunities and health for women and their children [24], [106]. More specifically, the approach could be two-fold to ensure the healthy life for rural adolescent women, which includes – to delay the age of marriage among unmarried adolescents, by providing better information to the parents of unmarried girls in particular and community in general regarding other options/avenues in education and the economic sphere. In this connection, the role of Women Advocacy Group (WAG) and Self Help Groups (SHG) comprising adolescent women at the village level could be effective. On the other hand, support can be provided to adolescent married women through targeted interventions that include working with the husband and in-laws in order to delay childbearing, promotion of contraceptive use and aware them not to link early childbirth with the honor of the family.

In the light of the above discussion, future policies and programs must not only address young people as individuals but consider them in the context of their overall development. In this regard special efforts must be made by the Department of Women and Child Development and Department of Youth to encourage effective participation of young adults in civil society and decision making processes. This study also emphasizes the importance of the recent law enforcement on the Prohibition of Child Marriage Act-2006 [107] which restricts minimum age at marriage to 21 years for boys and 18 years for girls by integrating *panchayat's* (local self-government body at the village level) accountability towards effective implementation. Finally, the approach to the health needs of young married adolescents must not be from the viewpoint of problems to be solved and health problems to be addressed, rather, it must be recognised as a matter of right and a means to achieve the Millennium Development Goals [108].
